# Trends and Predictors of Glycemic Control Among Adults With Type 2 Diabetes Covered by Alabama Medicaid, 2011–2019

**DOI:** 10.5888/pcd20.220332

**Published:** 2023-09-14

**Authors:** Caroline A. Presley, Yulia Khodneva, Lucia D. Juarez, Carrie R. Howell, April A. Agne, Kevin R. Riggs, Lei Huang, Maria Pisu, Emily B. Levitan, Andrea L. Cherrington

**Affiliations:** 1 Division of Preventive Medicine, Department of Medicine, University of Alabama at Birmingham Heersink School of Medicine; 2 Department of Epidemiology, School of Public Health, University of Alabama at Birmingham

## Abstract

**Introduction:**

Despite advances in diabetes management, only one-quarter of people with diabetes in the US achieve optimal targets for glycated hemoglobin A_1c_ (HbA_1c_), blood pressure, and cholesterol. We sought to evaluate temporal trends and predictors of achieving glycemic control among adults with type 2 diabetes covered by Alabama Medicaid from 2011 through 2019.

**Methods:**

We completed a retrospective analysis of Medicaid claims and laboratory data, using person-years as the unit of analysis. Inclusion criteria were being aged 19 to 64 years, having a diabetes diagnosis, being continuously enrolled in Medicaid for a calendar year and preceding 12 months, and having at least 1 HbA_1c_ result during the study year. Primary outcomes were HbA_1c_ thresholds of <7% and <8%. Primary exposure was study year. We conducted separate multivariable-adjusted logistic regressions to evaluate relationships between study year and HbA_1c_ thresholds.

**Results:**

We included 43,997 person-year observations. Mean (SD) age was 51.0 (9.9) years; 69.4% were women; 48.1% were Black, 42.9% White, and 0.4% Hispanic. Overall, 49.1% had an HbA_1c_ level of <7% and 64.6% <8%. Later study years and poverty-based eligibility were associated with lower probability of reaching target HbA_1c_ levels of <7% or <8%. Sex, race, ethnicity, and geography were not associated with likelihood of reaching HbA_1c_ <7% or <8% in any model.

**Conclusion:**

Later study years were associated with lower likelihood of meeting target HbA_1c_ levels compared with 2011, after adjusting for covariates. With approximately 35% not meeting an HbA_1c_ target of <8%, more work is needed to improve outcomes of low-income adults with type 2 diabetes.

**Summary**

**What is already known on this topic?**

Alabama has the third highest prevalence of diabetes in the US. Despite advances in diabetes management, only one-quarter of people with diabetes in the US achieve optimal target levels for glycated hemoglobin A_1c_, blood pressure, and cholesterol, which are key to preventing diabetes complications, illness, and death.

**What is added by this report?**

In our study, adults covered by Alabama Medicaid had levels of glycemic control that were similar to those of nationally representative samples; we found no differences by race, ethnicity, or geography. More than one-third did not achieve target glycemic control.

**What are the implications for public health practice?**

More work is needed, including potential Medicaid policy changes, to support people with diabetes covered by Medicaid.

## Introduction

The prevalence of diabetes in the US increased during the past 2 decades (1). The prevalence of diabetes also increased during this period in Alabama; in 2019, 14% of adults had diabetes, ranking third among the states, up from 8.4% in 2004 (2). Although the scientific knowledge related to diabetes management, including lifestyle modification and pharmacologic treatments, has advanced considerably, approximately one-quarter of people with diagnosed diabetes in the US achieve optimal target levels for glycated hemoglobin A_1c_ (HbA_1c_), blood pressure, and cholesterol (1,3–5). Diabetes is the number one cause of kidney failure, new cases of blindness, and nontraumatic lower limb amputation in the US (1). Care for people with diabetes accounts for more than 1 in 4 US health care dollars spent (6).

Prior studies evaluated rates of glycemic control and demographic and clinical factors associated with achieving optimal control among adults with diabetes, defined as an HbA_1c_ level below an individualized target ranging from 7% to 8.5% (4,5). Two recent studies using National Health and Nutrition Examination Survey (NHANES) data reported temporal trends in levels of optimal glycemic control among adults with diabetes. One study demonstrated lower odds of an HbA_1c_ below an individualized target in 2014–2016 compared with 2005–2008, and another reported a lower proportion of people reaching both stringent and less stringent HbA_1c_ targets in 2015–2018 compared with 2007–2010 (4,5). Younger age, racial and ethnic minority status, and lack of health insurance were factors associated with being less likely to achieve optimal glycemic control (4).

Although the aforementioned studies used nationally representative samples, health insurance coverage (ie, commercial, Medicare, Medicaid) was not evaluated as a factor related to glycemic control beyond the presence or absence of coverage, and levels of optimal glycemic control were not described for the different coverage groups. Dall et al showed similar levels of diabetes control by insurance type in a national sample in 2012 (7). Another study found a higher proportion of controlled diabetes among commercially insured adults than among adults covered by Medicare or Medicaid in a national sample from 2011 and 2012. In Alabama, 71% of adults with commercial insurance had controlled diabetes, 55% with Medicare, and 52% with Medicaid (8). These prior studies of diabetes outcomes in populations covered by Medicaid have been limited by the use of diagnosis codes to define “controlled diabetes” instead of HbA_1c_ laboratory results.

Populations with low incomes (<200% of federal poverty level) are at a particularly high risk for poor diabetes outcomes, including diabetes complications (9–12). In Alabama, 36% of the population has a low income; in 2019, one in 8 adults aged 18 to 64 years in Alabama was covered by Medicaid (13). Given these factors, Alabama has a high burden of diabetes complications and death. Given the limitations of prior studies of glycemic control in Medicaid or low-income populations and the lack of data on temporal trends in these populations, we sought to evaluate temporal changes from 2011 through 2019 in achieving glycemic control — using laboratory data and HbA_1c_ targets of <7% and <8% — among adults with type 2 diabetes who are Medicaid beneficiaries in Alabama.

## Methods

We conducted a retrospective analysis of Medicaid claims and laboratory data as part of an observational study of the quality of care of adults with diabetes covered by Alabama Medicaid. Medicaid eligibility for adults in Alabama includes parents of minor children with incomes 18% or less of the federal poverty level and adults with disability eligible for the Supplemental Security Income program (13). Enrollment and claims data for people with diabetes were provided for research purposes by the Alabama Medicaid Agency. HbA_1c_ values for Alabama Medicaid beneficiaries were provided for research purposes by LabCorp and Quest Diagnostics. Data use agreements do not allow for sharing of this information. All procedures performed in studies involving human participants were in accordance with the ethical standards of the institutional review board at the University of Alabama at Birmingham.

**Figure 1:**
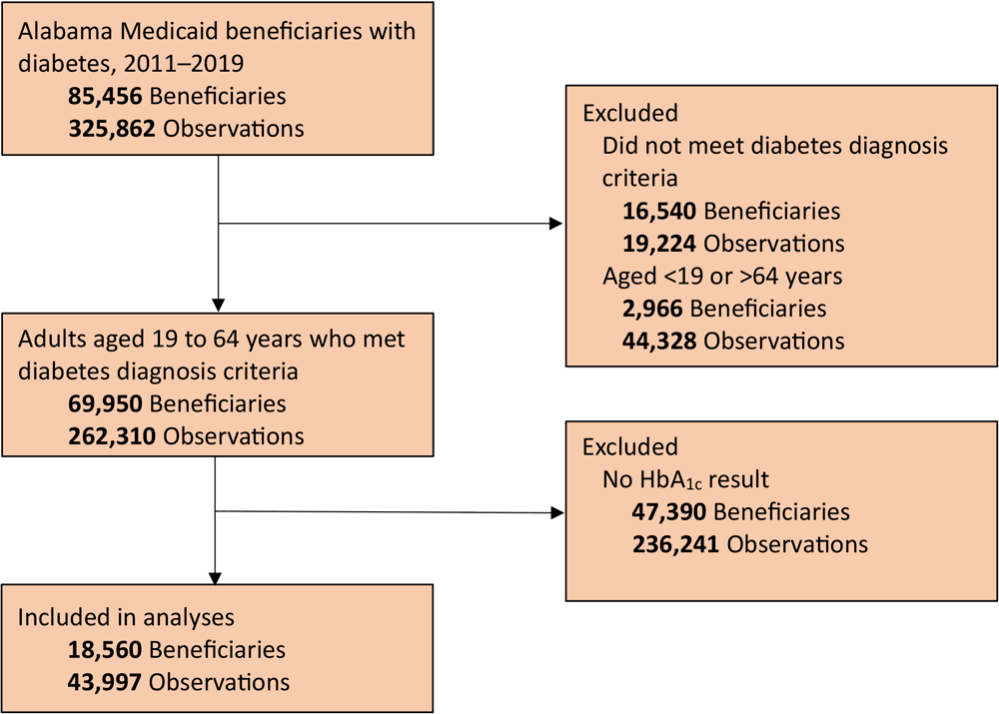
Flow diagram of included observations of adults with type 2 diabetes covered by Alabama Medicaid, 2011–2019.

The unit of analysis was person-year observation. In other words, each calendar year that a person met all of the inclusion criteria was considered as a data point, with people potentially contributing multiple data points for each year from 2011 through 2019. Inclusion criteria were 1) being aged 19 to 64 years; 2) having a diagnosis of diabetes (defined by the Chronic Conditions Data Warehouse as having ≥1 inpatient, skilled nursing facility, or home health agency claim or 2 outpatient claims with valid diagnosis codes in the prior 2 years) (14); 3) being continuously enrolled in Alabama Medicaid for a 12-month calendar year and the prior year; and 4) having at least 1 HbA_1c_ result during 2011–2019. For people with more than 1 HbA_1c_ value in a calendar year, we selected the earliest value for analysis. We included 43,997 person-year observations (Figure 1), with 4,022 to 5,471 observations per study year.

### Measures

Our primary outcomes were based on the first HbA_1c_ result for a person in the study year; we used HbA_1c_ thresholds of <7% and <8%. HbA_1c_, which represents the average blood glucose level for a person in the preceding 3 months, provides an estimate of chronic glycemia and the efficacy of a person’s diabetes treatment regimen. An HbA_1c_ target of <7% is a stringent target for the age group included in this study; an HbA_1c_ of <8% is the less stringent target for those in this age group with comorbidities, complications, or risk of hypoglycemia (3). We obtained laboratory results for Alabama Medicaid beneficiaries from 2011 through 2019 from LabCorp and Quest Diagnostics. Medicaid claims data show that these companies provide approximately 45% of all laboratory testing for Medicaid beneficiaries. Laboratory data were matched to Medicaid beneficiaries through linkage of first name, last name, and birth date. Our primary exposure was the study year of the HbA_1c_ result from 2011 through 2019.

### Covariates

We adjusted for demographic and clinical factors from Medicaid claims data shown to be associated with glycemic control (15,16). Demographic information included age, sex, race and ethnicity (based on Medicaid enrollment data), reason for Medicaid eligibility, and geography (urban, small town, or rural, based on zip code). Clinical data included insulin use, ambulatory care visits, acute care utilization, and medical comorbidities. Insulin use was based on the National Drug Code recorded on pharmacy claims (17). We determined a participant to be using insulin if their data showed an insulin fill up to the month before the HbA_1c_ result. We included ambulatory care visits in the year before the HbA_1c_ result, including total number of primary care visits and the presence of 1 or more endocrinology visits. We included acute care use based on the presence of 1 or more hospitalizations or the presence of 1 or more emergency department visits. We assessed the presence of 16 medical comorbidities that make up the Charlson Comorbidity Index by examining diagnoses recorded on claims in the year before the HbA_1c_ result (18). 

### Analysis

We completed descriptive statistics to characterize the study sample. We generated means and SDs for HbA_1c_ levels by study year. We then calculated the sample size and percentage of people with HbA_1c_ <7% and <8% by study year. We conducted separate multivariable logistic regressions to evaluate the relationship between study year and an HbA_1c_ result: <7% and <8%. Model 1 was unadjusted; model 2 was adjusted for demographic factors (age, sex, race and ethnicity, reason for eligibility, geography); model 3 was adjusted for demographic factors plus clinical factors (insulin use, ambulatory care use, acute care utilization, and medical comorbidities). We conducted secondary analyses that repeated the same models above for each HbA_1c_ result with study year included as a continuous variable. The models used the generalized estimating equation approach to account for people contributing multiple calendar year observations. 

In an additional analysis, we compared our study sample to the sample of Medicaid beneficiaries with diabetes (N = 262,310 person-years) and the subset of the larger sample with diabetes who had a claim for an HbA_1c_ test but not an HbA_1c_ result (n = 149,647) to assess for potential bias. 

We conducted all statistical analyses in SAS version 9.3.1 (SAS Institute Inc).

## Results

The mean (SD) age of the Alabama Medicaid beneficiaries with diabetes in our analysis was 51.0 (9.9) years; 69.4% were women; 48.1% were Black and 42.9% were White (Table 1). Based on zip code, 67.8% lived in an urban area, 14.4% in a small town, and 17.9% in a rural area. More than three-fourths were eligible for Medicaid based on disability (77.7%); 22.3% were eligible based on poverty, with an increasing proportion based on poverty in later study years. One-third used insulin, and 28.0% had diabetes with complications. The percentage of beneficiaries who had diabetes with complications increased to 36.7% in 2019.

**Table 1:** Characteristics of Medicaid Beneficiaries With Diabetes in Alabama, by Study Year, 2011–2019^a^

Characteristics	Overall	2011	2012	2013	2014	2015	2016	2017	2018	2019
**No.**	43,997	4,022	4,764	5,252	5,031	4,725	5,014	5,294	5,471	4,424
**HbA_1c, _mean (SD), %**	7.72 (2.24)	7.63 (2.18)	7.72 (2.26)	7.75 (2.23)	7.75 (2.24)	7.79 (2.23)	7.83 (2.22)	7.85 (2.27)	7.48 (2.37)	7.64 (2.09)
**Age, mean (SD), y**	51.0 (9.9)	50.9 (9.9)	50.6 (10.0)	51.0 (9.9)	51.0 (9.8)	51.1 (9.8)	51.2 (9.8)	51.4 (9.8)	51.1 (10.1)	51.0 (10.0)
**Sex, no. (%)**										
Male	13,485 (30.6)	1,097 (27.3)	1,431 (30.0)	1,582 (30.1)	1,565 (31.1)	1,471 (31.1)	1,586 (31.6)	1,646 (31.1)	1,735 (31.7)	1,372 (31.0)
Female	30,512 (69.4)	2,925 (72.7)	3,333 (70.0)	3,670 (69.9)	3,466 (68.9)	3,254 (68.9)	3,428 (68.4)	3,648 (68.9)	3,736 (68.3)	3,052 (69.0)
**Race and ethnicity, no. (%)**										
Black	21,141 (48.1)	2,049 (50.9)	2,418 (50.8)	2,617 (49.8)	2,460 (48.9)	2,267 (48.0)	2,398 (47.8)	2,414 (45.6)	2,481 (45.3)	2,037 (46.0)
Hispanic	184 (0.4)	15 (0.4)	21 (0.4)	23 (0.4)	11 (0.2)	15 (0.3)	17 (0.3)	23 (0.4)	35 (0.6)	24 (0.5)
White	18,892 (42.9)	1,693 (42.1)	1,999 (42.0)	2,225 (42.4)	2,170 (43.1)	2,044 (43.3)	2,188 (43.6)	2,341 (44.2)	2,371 (43.3)	1,861 (42.1)
Other^b^	3,780 (8.6)	265 (6.6)	326 (6.8)	387 (7.4)	390 (7.8)	399 (8.4)	411 (8.2)	516 (9.7)	584 (10.7)	502 (11.3)
**Geography, no. (%)**										
Urban	29,816 (67.8)	2,702 (67.2)	3,165 (66.4)	3,537 (67.3)	3,412 (67.8)	3,249 (68.8)	3,472 (69.2)	3,629 (68.5)	3,719 (68.0)	2,931 (66.3)
Small town	6,318 (14.4)	647 (16.1)	775 (16.3)	801 (15.3)	782 (15.5)	711 (15.0)	677 (13.5)	695 (13.1)	676 (12.4)	554 (12.5)
Rural	7,863 (17.9)	673 (16.7)	824 (17.3)	914 (17.4)	837 (16.6)	765 (16.2)	865 (17.3)	970 (18.3)	1,076 (19.7)	939 (21.2)
**Eligibility reason, no. (%)**										
Disability	34,197 (77.7)	3,511 (87.3)	3,986 (83.7)	4,324 (82.3)	4,038 (80.3)	3,750 (79.4)	3,805 (75.9)	3,858 (72.9)	3,888 (71.1)	3,037 (68.6)
Poverty	9,800 (22.3)	511 (12.7)	778 (16.3)	928 (17.7)	993 (19.7)	975 (20.6)	1,209 (24.1)	1,436 (27.1)	1,583 (28.9)	1,387 (31.4)
**Insulin use in prior year, no. (%)**	14,654 (33.3)	1,403 (34.9)	1,649 (34.6)	1,860 (35.4)	1,722 (34.2)	1,569 (33.2)	1,597 (31.9)	1,672 (31.6)	1,753 (32.0)	1,429 (32.3)
**Ambulatory care use**										
No. of primary care visits, mean (SD)	4.96 (2.95)	5.14 (2.87)	4.81 (2.88)	4.85 (2.96)	4.91 (2.85)	4.88 (2.97)	5.01 (3.06)	5.11 (3.00)	4.99 (3.01)	4.94 (2.94)
≥1 Primary care visit, no. (%) of beneficiaries	41,363 (94.0)	3,786 (94.1)	4,457 (93.6)	4,909 (93.5)	4,771 (94.8)	4,432 (93.8)	4,728 (94.3)	4,988 (94.2)	5,133 (93.8)	4,159 (94.0)
No. of endocrinology visits, mean (SD)	0.10 (0.56)	0.07 (0.41)	0.08 (0.48)	0.10 (0.56)	0.10 (0.55)	0.10 (0.55)	0.12 (0.62)	0.11 (0.61)	0.11 (0.58)	0.11 (0.58)
≥1 Endocrinology visit, no. (%) of beneficiaries	2,025 (4.6)	142 (3.5)	185 (3.9)	238 (4.5)	226 (4.5)	217 (4.6)	257 (5.1)	250 (4.7)	289 (5.3)	221 (5.0)
**Acute care use, no. (%)**										
≥1 Emergency department visit	25,991 (59.1)	2,305 (57.3)	2,719 (57.1)	3,014 (57.4)	2,960 (58.8)	2,800 (59.3)	2,969 (59.2)	3,223 (60.9)	3,323 (60.7)	2,678 (60.5)
≥1 Hospitalization	13,141 (29.9)	1,297 (32.2)	1,387 (29.1)	1,584 (30.2)	1,490 (29.6)	1,433 (30.3)	1,465 (29.2)	1,571 (29.7)	1,611 (29.4)	1,303 (29.5)
**Medical comorbidities, no. (%)**										
Myocardial infarction	1,158 (2.6)	89 (2.2)	110 (2.3)	150 (2.9)	121 (2.4)	123 (2.6)	147 (2.9)	158 (3.0)	159 (2.9)	101 (2.3)
Congestive heart failure	5,929 (13.5)	562 (14.0)	605 (12.7)	703 (13.4)	657 (13.1)	658 (13.9)	696 (13.9)	695 (13.1)	747 (13.7)	606 (13.7)
Peripheral vascular disease	4,022 (9.1)	417 (10.4)	443 (9.3)	484 (9.2)	448 (8.9)	445 (9.4)	453 (9.0)	434 (8.2)	490 (9.0)	408 (9.2)
Cerebrovascular disease	4,467 (10.2)	412 (10.2)	449 (9.4)	500 (9.5)	535 (10.6)	504 (10.7)	539 (10.7)	526 (9.9)	566 (10.3)	436 (9.9)
Dementia	449 (1.0)	36 (0.9)	36 (0.8)	41 (0.8)	40 (0.8)	50 (1.1)	53 (1.1)	67 (1.3)	68 (1.2)	58 (1.3)
Chronic pulmonary disease	14,343 (32.6)	1,344 (33.4)	1,431 (30.0)	1,744 (33.2)	1,648 (32.8)	1,585 (33.5)	1,730 (34.5)	1,723 (32.5)	1,772 (32.4)	1,366 (30.9)
Rheumatic disease	1,261 (2.9)	116 (2.9)	120 (2.5)	141 (2.7)	147 (2.9)	144 (3.0)	142 (2.8)	159 (3.0)	173 (3.2)	119 (2.7)
Peptic ulcers	781 (1.8)	95 (2.4)	77 (1.6)	85 (1.6)	91 (1.8)	99 (2.1)	87 (1.7)	89 (1.7)	78 (1.4)	80 (1.8)
Diabetes with complication	12,316 (28.0)	922 (22.9)	1,088 (22.8)	1,145 (21.8)	1,184 (23.5)	1,190 (25.2)	1,486 (29.6)	1,782 (33.7)	1,894 (34.6)	1,625 (36.7)
Paraplegia and hemiplegia	788 (1.8)	64 (1.6)	80 (1.7)	91 (1.7)	90 (1.8)	90 (1.9)	99 (2.0)	97 (1.8)	93 (1.7)	84 (1.9)
Chronic renal failure	4,563 (10.4)	358 (8.9)	424 (8.9)	475 (9.0)	466 (9.3)	465 (9.8)	570 (11.4)	610 (11.5)	662 (12.1)	533 (12.0)
Mild liver disease	2,454 (5.6)	123 (3.1)	142 (3.0)	169 (3.2)	176 (3.5)	169 (3.6)	264 (5.3)	472 (8.9)	523 (9.6)	416 (9.4)
Moderate-severe liver disease	299 (0.7)	34 (0.8)	32 (0.7)	38 (0.7)	39 (0.8)	26 (0.6)	24 (0.5)	46 (0.9)	38 (0.7)	22 (0.5)
Any malignancy	1,892 (4.3)	180 (4.5)	223 (4.7)	237 (4.5)	202 (4.0)	217 (4.6)	219 (4.4)	205 (3.9)	227 (4.1)	182 (4.1)
Metastatic solid tumor	203 (0.5)	19 (0.5)	22 (0.5)	22 (0.4)	21 (0.4)	25 (0.5)	21 (0.4)	22 (0.4)	27 (0.5)	24 (0.5)
AIDS	420 (1.0)	34 (0.8)	32 (0.7)	45 (0.9)	48 (1.0)	49 (1.0)	38 (0.8)	62 (1.2)	62 (1.1)	50 (1.1)
**HbA_1c_ target, %**										
<7%	21,581 (49.1)	2,073 (51.5)	2,400 (50.4)	2,558 (48.7)	2,478 (49.3)	2,239 (47.4)	2,360 (47.1)	2,484 (46.9)	2,816 (51.5)	2,173 (49.1)
<8%	28,386 (64.6)	2,715 (67.5)	3,130 (65.7)	3,382 (64.4)	3,213 (63.9)	2,980 (63.1)	3,172 (63.3)	3,274 (61.8)	3,652 (66.8)	2,868 (64.8)

Abbreviation: HbA_1c_, glycated hemoglobin A_1c_.

a Data source: Enrollment and claims data for people with diabetes were provided for research purposes by the Alabama Medicaid Agency. HbA_1c_ values for Alabama Medicaid beneficiaries were provided for research purposes by LabCorp and Quest Diagnostics. Unit of analysis was person-years.

b Includes Asian or Pacific Islander, American Indian or Alaskan Native, Other Race or Ethnicity, or Unknown/Not Provided.

**Figure 2:**
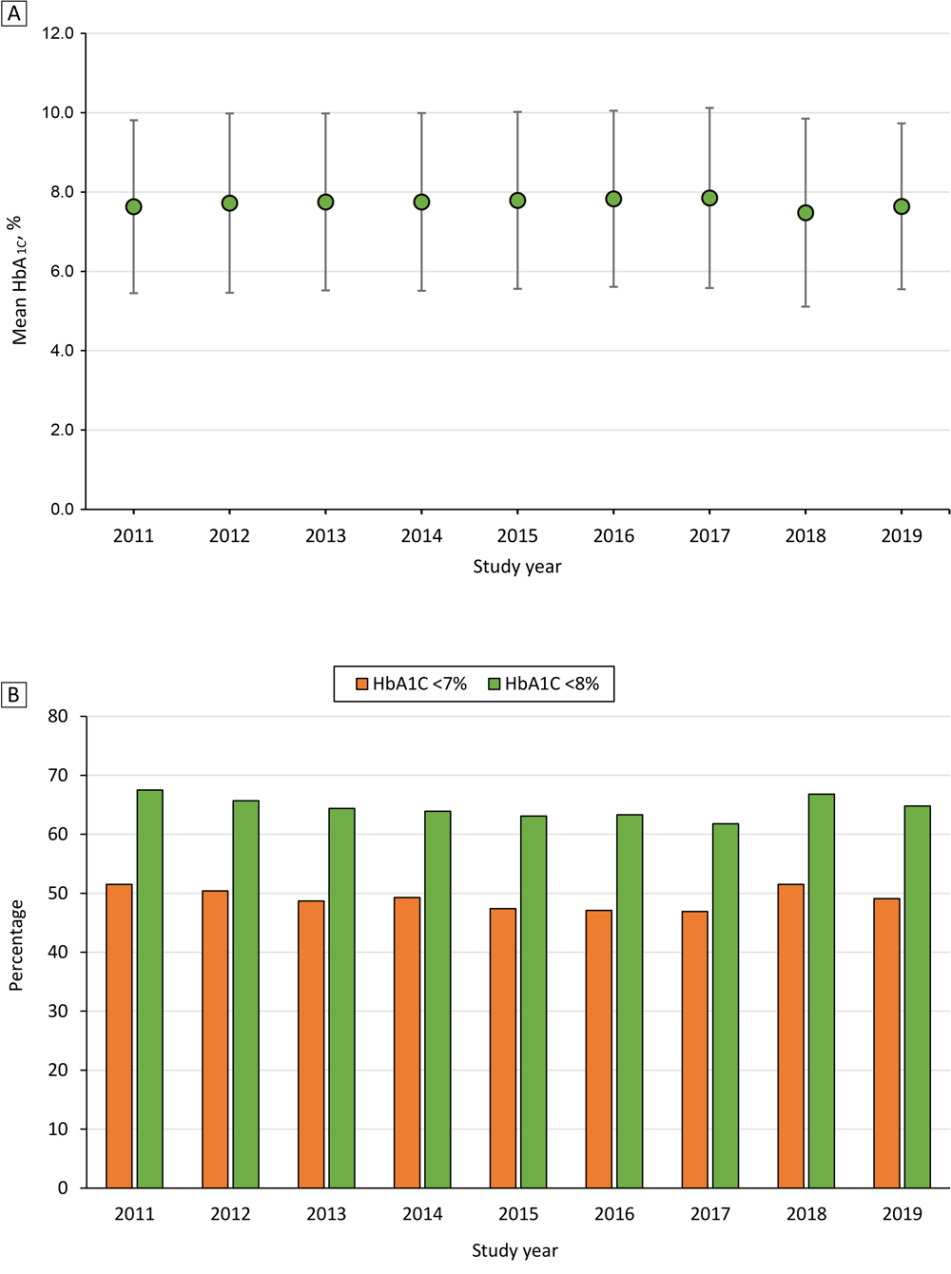
Mean HbA_1c_ by study year and glycemic control among Medicaid beneficiaries with diabetes in Alabama, 2011–2019. A, Mean HbA_1c_ by study year. Error bars indicate SDs. B, Percentage of beneficiaries with an HbA_1c_ <7% and <8%. The unit of analysis was person-years.

Mean (SD) HbA_1c_ for the study period was 7.72% (2.24%); it ranged from 7.63% in 2011 to 7.85% in 2017 (Table 1, Figure 2). For the stricter HbA_1c_ target, 49.1% had an HbA_1c_ result <7%, ranging from 46.9% in 2017 to 51.5% in 2011 and 2018. For the higher HbA_1c_ target, 64.6% had an HbA_1c_ result <8%, ranging from 61.8% in 2017 to 67.5% in 2011. Of the 9 study years, the smallest proportion of the sample with an HbA_1c _at the target level was found in 2017 (46.9% with a result <7% and 61.8% with a result <8%).

**Table:** 

Graph	Study year								
	2011	2012	2013	2014	2015	2016	2017	2018	2019
**A. HbA_1c_ Mean (SD), %**	7.63 (2.18)	7.72 (2.26)	7.75 (2.23)	7.75 (2.24)	7.79 (2.23)	7.83 (2.22)	7.85 (2.27)	7.48 (2.37)	7.64 (2.09)
**B. Glycemic control**									
Percentage of beneficiaries with HbA1c <7%	51.5	50.4	48.7	49.3	47.4	47.1	46.9	51.5	49.1
Percentage of beneficiaries with HbA1c <8%	67.5	65.7	64.4	63.9	63.1	63.3	61.8	66.8	64.8

In models testing the association of study year with an HbA_1c_ result <7% that included demographic and clinical factors as covariates, the study years of 2013–2017 and 2019 were associated with lower odds of an HbA_1c_ result <7% compared with 2011 (Table 2). Poverty-based eligibility was significantly associated with lower odds of having an HbA_1c_ result <7% (odds ratio [OR] = 0.87; 95% CI, 0.84–0.89). We found similar results in the logistic regression models that tested the association of study year with an HbA_1c_ result <8% (Table 3); years 2012–2017 and 2019 were associated with lower odds of an HbA_1c_ result <8% compared with 2011. In these models, older age was associated with higher odds of having an HbA_1c_ result <8%; for every 10-year increment in age, the likelihood of achieving glycemic control increased by 3% (OR = 1.03; 95% CI, 1.02–1.04). Sex, race, ethnicity, and geography were not associated with odds of an HbA_1c_ result <7% or <8% in any of the models. Of the 16 comorbidities assessed as part of the Charlson Comorbidity Index, diabetes with complication was the only covariate associated with lower likelihood of achieving an HbA_1c_ result <7% or <8%. Several comorbidities were associated with higher odds of an HbA_1c_ result <7% or <8%, including cerebrovascular disease, dementia, chronic obstructive pulmonary disease, peptic ulcer disease, chronic renal failure, and AIDS. For the secondary analyses, which included study year as a continuous variable, study year did not have a significant linear association with an HbA_1c_ result <7% or <8% (OR = 0.99; 95% CI, 0.99–1.00 in both models).

**Table 2:** Characteristics Associated With Having an HbA_1c_ <7% Among Medicaid Beneficiaries With Diabetes in Alabama, 2011–2019^a^

Characteristic	Unadjusted OR (95% CI)	OR (95% CI), adjusted for demographic factors^b^	OR (95% CI), adjusted for demographic and clinical factors^c^
**Year**			
2011	1 [Reference]	1 [Reference]	1 [Reference]
2012	0.98 (0.96–1.01)	0.98 (0.96–1.01)	0.99 (0.97–1.02)
2013	0.93 (0.90–0.96)	0.93 (0.90–0.96)	0.95 (0.92–0.98)
2014	0.94 (0.91–0.97)	0.94 (0.91–0.96)	0.96 (0.93–0.99)
2015	0.90 (0.87–0.93)	0.89 (0.87–0.92)	0.91 (0.89–0.95)
2016	0.88 (0.85–0.91)	0.88 (0.85–0.90)	0.90 (0.87–0.93)
2017	0.87 (0.84–0.90)	0.86 (0.84–0.89)	0.90 (0.87–0.93)
2018	0.94 (0.91–0.97)	0.93 (0.90–0.96)	0.99 (0.95–1.02)
2019	0.90 (0.86–0.93)	0.89 (0.86–0.92)	0.95 (0.92–0.99)
**Age (per 10 year)**	1.02 (1.00–1.03)	1.03 (1.01–1.04)	1.00 (0.99–1.02)
**Sex**			
Male	1 [Reference]	1 [Reference]	1 [Reference]
Female	0.97 (0.95–1.00)	0.98 (0.95–1.01)	0.99 (0.97–1.02)
**Race and ethnicity**			
Black	0.95 (0.93–0.98)	0.95 (0.93–0.98)	0.99 (0.96–1.01)
Hispanic	0.77 (0.61–0.96)	0.77 (0.62–0.97)	0.85 (0.68–1.06)
White	1 [Reference]	1 [Reference]	1 [Reference]
Other^d^	0.96 (0.92–1.01)	0.96 (0.91–1.00)	0.97 (0.93–1.02)
**Geography**			
Urban	1 [Reference]	1 [Reference]	1 [Reference]
Small town	0.98 (0.95–1.01)	0.98 (0.95–1.01)	0.97 (0.94–1.00)
Rural	0.99 (0.96–1.03)	0.99 (0.95–1.02)	0.98 (0.95–1.01)
**Reason for eligibility**			
Disability	1 [Reference]	1 [Reference]	1 [Reference]
Poverty	0.95 (0.93–0.98)	0.97 (0.95–1.00)	0.87 (0.84–0.89)
Insulin use	0.44 (0.43–0.46)	—	0.44 (0.42–0.45)
**Ambulatory care use in prior year**			
Internist visits (per visit)	1.01 (1.00–1.01)	—	1.01 (1.01–1.01)
Endocrinology visits (yes/no)	0.84 (0.80–0.88)	—	0.92 (0.87–0.97)
**Acute care use in prior year**			
Any ED visit (yes/no)	0.99 (0.97–1.00)	—	0.99 (0.98–1.01)
Any hospitalization (yes/no)	1.00 (0.98–1.01)	—	1.02 (1.01–1.04)
**Medical comorbidities in prior year**			
Myocardial infarction	0.98 (0.93–1.03)	—	0.99 (0.94–1.05)
Congestive heart failure	0.99 (0.96–1.02)	—	1.00 (0.97–1.03)
Peripheral vascular disease	1.01 (0.98–1.04)	—	1.02 (0.99–1.05)
Cerebrovascular disease	1.03 (1.01–1.06)	—	1.04 (1.01–1.07)
Dementia	1.11 (1.02–1.21)	—	1.16 (1.06–1.27)
Chronic pulmonary disease	1.07 (1.05–1.09)	—	1.06 (1.04–1.08)
Rheumatic disease	1.02 (0.97–1.08)	—	1.03 (0.98–1.08)
Peptic ulcers	1.09 (1.03–1.15)	—	1.09 (1.04–1.15)
Diabetes with complication	0.87 (0.85–0.89)	—	0.90 (0.88–0.92)
Paraplegia and hemiplegia	1.03 (0.97–1.09)	—	1.01 (0.95–1.08)
Chronic renal failure	0.99 (0.97–1.03)	—	1.07 (1.03–1.10)
Mild liver disease	1.01 (0.97–1.04)	—	1.03 (0.99–1.07)
Moderate-severe liver disease	1.03 (0.94–1.14)	—	1.06 (0.95–1.17)
Any malignancy	1.09 (0.97–1.22)	—	1.06 (0.94–1.19)
Metastatic solid tumor	1.01 (0.97–1.06)	—	0.99 (0.95–1.04)
AIDS	1.15 (1.04–1.28)	—	1.12 (1.02–1.23)

Abbreviations: — , does not apply; HbA_1c_, glycated hemoglobin A_1c_.

a Data source: Enrollment and claims data for people with diabetes were provided for research purposes by the Alabama Medicaid Agency. HbA_1c_ values for Alabama Medicaid beneficiaries were provided for research purposes by LabCorp and Quest Diagnostics.

b Adjusted for demographic factors (age, sex, race and ethnicity, reason for eligibility, geography).

c Adjusted for demographic and clinical factors (insulin use, ambulatory care use, acute care utilization, and medical comorbidities). Models used generalized estimating equations to account for people contributing multiple calendar year observations.

d Includes Asian or Pacific Islander, American Indian or Alaskan Native, other race or ethnicity, or unknown/not provided.

**Table 3:** Characteristics Associated With Having an HbA_1c_ <8% Among Medicaid Beneficiaries With Diabetes in Alabama, 2011–2019^a^

Characteristic	Unadjusted OR (95% CI)	OR (95% CI), adjusted for demographic factors^b^	OR (95% CI), adjusted for demographic and clinical factors^c^
**Year**			
2011	1 [Reference]	1 [Reference]	1 [Reference]
2012	0.97 (0.95–0.99)	0.97 (0.95–0.99)	0.98 (0.96–1.00)
2013	0.95 (0.92–0.97)	0.94 (0.92–0.96)	0.96 (0.94–0.98)
2014	0.94 (0.92–0.96)	0.93 (0.91–0.95)	0.95 (0.93–0.97)
2015	0.92 (0.90–0.95)	0.91 (0.89–0.94)	0.93 (0.91–0.96)
2016	0.92 (0.89–0.94)	0.90 (0.88–0.93)	0.93 (0.90–0.95)
2017	0.89 (0.87–0.91)	0.88 (0.85–0.90)	0.90 (0.88–0.93)
2018	0.95 (0.93–0.97)	0.93 (0.91–0.96)	0.97 (0.95–1.00)
2019	0.92 (0.90–0.95)	0.91 (0.88–0.93)	0.95 (0.93–0.98)
**Age** **(per 10 year)**	1.05 (1.04–1.06)	1.05 (1.04–1.06)	1.03 (1.02–1.04)
**Sex**			
Male	1 [Reference]	1 [Reference]	1 [Reference]
Female	0.98 (0.96–1.00)	0.98 (0.96–1.00)	0.99 (0.97–1.01)
**Race and ethnicity**			
Black	0.97 (0.95–0.99)	0.97 (0.95–0.99)	1.00 (0.98–1.02)
Hispanic	0.89 (0.76–1.04)	0.90 (0.77–1.04)	0.96 (0.84–1.11)
White	1 [Reference]	1 [Reference]	1 [Reference]
Other^d^	0.98 (0.95–1.01)	0.97 (0.94–1.01)	0.99 (0.96–1.02)
**Geography**			
Urban	1 [Reference]	1 [Reference]	1 [Reference]
Small town	1.00 (0.97–1.02)	0.99 (0.97–1.02)	0.98 (0.96–1.01)
Rural	1.00 (0.98–1.03)	0.99 (0.97–1.02)	0.99 (0.97–1.01)
**Reason for eligibility**			
Disability	1 [Reference]	1 [Reference]	1 [Reference]
Poverty	0.98 (0.96–1.00)	1.00 (0.98–1.02)	0.91 (0.89–0.93)
Insulin use	0.56 (0.55–0.57)	—	0.55 (0.54–0.57)
**Ambulatory care use in prior year**			
Internist visits (per visit)	1.01 (1.01–1.01)	—	1.01 (1.01–1.01)
Endocrinology visits (yes/no)	0.86 (0.83–0.90)	—	0.94 (0.90–0.98)
**Acute care use in prior year**			
Any emergency department visit (yes/no)	0.98 (0.97–0.99)	—	0.99 (0.98–1.00)
Any hospitalization (yes/no)	0.99 (0.97–1.00)	—	1.01 (0.99–1.02)
**Medical comorbidities in prior year**			
Myocardial infarction	0.99 (0.95–1.03)	—	1.00 (0.96–1.04)
Congestive heart failure	1.00 (0.98–1.03)	—	1.01 (0.99–1.03)
Peripheral vascular disease	1.02 (1.00–1.04)	—	1.02 (0.99–1.04)
Cerebrovascular disease	1.02 (1.00–1.04)	—	1.02 (1.00–1.04)
Dementia	1.10 (1.04–1.17)	—	1.13 (1.06–1.20)
Chronic pulmonary disease	1.05 (1.04–1.07)	—	1.04 (1.02–1.05)
Rheumatic disease	1.01 (0.97–1.05)	—	1.00 (0.96–1.04)
Peptic ulcers	1.02 (0.98–1.06)	—	1.01 (0.97–1.05)
Diabetes with complication	0.91 (0.90–0.93)	—	0.94 (0.92–0.95)
Paraplegia and hemiplegia	1.05 (1.00–1.10)	—	1.05 (1.00–1.10)
Chronic renal failure	1.02 (0.99–1.04)	—	1.07 (1.04–1.09)
Mild liver disease	1.02 (0.99–1.05)	—	1.04 (1.01–1.07)
Moderate-severe liver disease	1.07 (0.99–1.14)	—	1.07 (0.99–1.14)
Any malignancy	1.05 (1.02–1.09)	—	1.03 (0.99–1.06)
Metastatic solid tumor	1.08 (1.00–1.17)	—	1.03 (0.95–1.12)
AIDS	1.10 (1.02–1.19)	—	1.07 (1.00–1.14)

Abbreviations: — , does not apply; HbA_1c_, glycated hemoglobin A_1c_.

a Data source: Enrollment and claims data for people with diabetes were provided for research purposes by the Alabama Medicaid Agency. HbA_1c_ values for Alabama Medicaid beneficiaries were provided for research purposes by LabCorp and Quest Diagnostics.

b Adjusted for demographic factors (age, sex, race/ethnicity, reason for eligibility, geography).

c Adjusted for demographic and clinical factors (insulin use, ambulatory care use, acute care utilization, and medical comorbidities). Models used generalized estimating equations to account for people contributing multiple calendar year observations.

d Includes Asian or Pacific Islander, American Indian or Alaskan Native, other race or ethnicity, or unknown/not provided.

In our assessment of potential bias, we found that our study sample was similar for most demographic and clinical factors to the sample of Medicaid beneficiaries with diabetes and the subset of the larger sample with diabetes who had a claim for an HbA_1c_ test but not an HbA_1c_ result (Appendix). We found modest differences: our study sample had a lower proportion of beneficiaries with poverty-based eligibility, a higher proportion in urban areas, a higher proportion who used insulin, and a higher level of primary care use.

## Discussion

In our study of adults with type 2 diabetes covered by Alabama Medicaid and represented by 43,997 person-year observations from 2011 through 2019, we found that just under half met an HbA_1c_ target of <7% and approximately 6 in 10 met an HbA_1c_ target of <8%. After adjusting for important covariates, later study years, with the exception of 2018, were associated with lower odds of meeting target HbA_1c_ levels, compared with the reference year of 2011. We found that Medicaid eligibility status was related to achieving optimal glycemic control — poverty-based eligibility, compared with disability-based eligibility, was associated with lower odds of achieving HbA_1c_ targets. We did not find differences by race or ethnicity for meeting HbA_1c_, a finding that differed from findings in prior studies that used NHANES data or primary care data (4,5,19). Additionally, clinical factors related to the severity of diabetes, including insulin use, endocrinology care, and diagnosis of diabetes complications were significantly associated with lower likelihood of achieving glycemic control. Several medical comorbidities were associated with a higher likelihood of achieving glycemic control, and various factors influenced these associations. Disease-specific factors, including altered glucose and insulin metabolism, may apply to chronic renal failure, while other changes to patient or provider behaviors related to control or increased overall contact with a health care system may apply across different comorbidities (20).

Overall, the proportion of adults meeting HbA_1c_ targets of <7% (49.1%) and <8% (64.6%) in our study were similar to the proportion reaching optimal glycemic control in studies of nationally representative samples with all types of health insurance coverage. For example, Kazemian et al found that 64% met an individualized HbA_1c_ target, while Fang et al found that 50.5% had an HbA_1c_ result <7% and 75.4% were <8% (4,5). Furthermore, the proportion meeting an HbA_1c_ target of <8% (64.6%) was higher in our study than in a prior study, which used diagnosis codes and found that 52% of Medicaid beneficiaries had controlled diabetes (vs 71% with commercial insurance) (7,8). A strength of our study was the use of HbA_1c_ laboratory results, instead of diagnosis codes, to assess glycemic control, which is in line with guideline recommendations to use HbA_1c_ as a measure of glycemic control (3).

We did not find differences in achieving optimal glycemic control between racial or ethnic groups or between those living in rural and urban areas. In prior studies, Black adults with diabetes, compared with non-Hispanic White adults, were less likely to receive recommended HbA_1c_ testing or other screening measures, and had higher average HbA_1c_ levels and higher rates of death (19,21–23). Similar disparities have been seen in comparisons of rural and urban areas: death rates were higher among adults with diabetes living in rural areas (23). Given the limited eligibility for Alabama Medicaid among adults, the lack of disparities by racial or ethnic group or geography may have resulted from similar resource constraints among the low-income population across these groups. However, we found that those with poverty-based eligibility had lower odds of reaching target HbA_1c_ levels than those with disability-based eligibility, suggesting that people in the Medicaid population with greater financial need may need additional resources to achieve glycemic control and improve diabetes-related clinical outcomes. These results are in line with those of other studies, which demonstrated that low socioeconomic status and other social determinants of health, including food insecurity, were linked to poor outcomes among people with diabetes, including increased mortality risk and worse self-management and glycemic control (24–26).

Our study results are consistent with the results of other studies that showed a lower likelihood of achieving HbA_1c_ targets in years 2014–2018 compared with years 2005–2010 (4,5). This trend occurred when guidelines shifted to recommending individualized HbA_1c_ targets based on age, presence of diabetes complications or comorbid conditions, and risk of hypoglycemia, which may be less stringent than previously recommended targets. The shift in guidelines was based on major trials showing the risks and benefits of uniform intensive glycemic control (27,28). Along with the trend in lower likelihood of achieving HbA_1c_ targets, the incidence of diabetes complications since 2010 among younger and middle-aged adults has been increasing, following a period of sustained decline (29). A cost-effectiveness study accounting for rates of diabetes complications showed that individualized HbA_1c_ targets are cost-effective and increase quality of life compared with uniform intensive glycemic control (30). Our study and others demonstrated that more than one-third of adults with diabetes continued to have suboptimal glycemic control even when accounting for less stringent individualized targets (4,5).

Alabama has not expanded Medicaid eligibility under the Affordable Care Act; an estimated 200,000 adults in Alabama, who would be eligible under expansion, remain uninsured (31). Forty states and Washington, DC, have extended Medicaid eligibility to nearly all adults with incomes up to 138% of the federal poverty level (13). Given our results, which show similar glycemic control among adults covered by Alabama Medicaid and nationally representative populations with various types of health insurance, Medicaid expansion in Alabama or other Medicaid policies may be an important route to improve diabetes care and outcomes in the state. In other states, Medicaid expansion or other state policies have influenced diabetes care, self-management, and outcomes. Medicaid-eligible people with diabetes in Medicaid expansion states were more likely than their counterparts in nonexpansion states to afford a physician visit, be seen by a physician, or receive diabetes medications and supplies within the previous year; no significant differences were seen between these groups for self-management of diabetes (32–35). Furthermore, Medicaid expansion has influenced the uptake of newer diabetes medications; increases in rates of use of noninsulin diabetes medications, including glucagon-like peptide 1 receptor agonists and sodium-glucose cotransporter-2 inhibitors, were significantly greater in expansion states than in nonexpansion states (36). Finally, in a study of Wisconsin Medicaid beneficiaries, a reduction in drug copayment amounts was associated with significantly increased medication adherence among adults with diabetes (37).

### Limitations

Our study has several limitations. Participant demographic characteristics, including race and ethnicity, were based on Medicaid enrollment data, not participant self-report. We were unable to account for diabetes self-management behaviors or other psychosocial factors that influence glycemic control. Our outcome was based on a single HbA_1c_ result, a snapshot of glycemic control for the preceding 3 months. We were unable to assess an individualized HbA_1c_ target, but we did evaluate 2 HbA_1c_ targets (<7% and <8%). For the age group included in this study (19–64 y), an HbA_1c_ result <8% is the recommended target for those with comorbidities, complications, or risk of hypoglycemia (3). We did not assess clinical outcomes or other recommended risk factor controls for adults with diabetes (ie, blood pressure and lipid control). People in our study sample were connected to the health care system; the proportion achieving glycemic control in our sample may be larger than the proportion of the Alabama Medicaid population with diabetes who did not receive HbA_1c_ testing or did not have HbA_1c_ values from Quest Diagnostics or LabCorp. We were able to obtain HbA_1c_ values only from LabCorp and Quest Diagnostics; we did not have HbA_1c_ values for tests performed in other laboratories. People with available HbA_1c_ values were similar to those with claims for HbA_1c_ testing but no available HbA_1c_ results, except that people with results were more likely to live in urban areas, to have disability-based eligibility, to use insulin, and to use primary care. Some of these factors were associated with higher odds of achieving glycemic control (disability-based eligibility, primary care use) and some were associated with lower odds (insulin use, urban residence). Because the magnitude of the differences was not large, we would not expect a substantial effect on the observed associations. Finally, because of restrictive Medicaid eligibility in Alabama, our study population was very low-income or had a disability; the proportion achieving glycemic control or the relationships between factors associated with glycemic control may not be generalizable to states that expanded Medicaid eligibility to include households up to 138% of the federal poverty level.

### Conclusions

Among adults covered by Alabama Medicaid, 49.1% met an HbA_1c_ target <7% and 64.6% met an HbA_1c_ target <8%. Later study years were associated with lower likelihood of meeting HbA_1c_ targets compared with 2011. These results were similar to studies of nationally representative samples that showed no differences across racial or ethnic groups or geography. Medicaid coverage is an important factor in allowing low-income adults with diabetes to access health care and achieve optimal glycemic control. With more than 35% of Alabama Medicaid–eligible adults with diabetes not meeting the less stringent HbA_1c_ target of <8%, more work is needed to translate scientific advances to improve the care and outcomes of low-income adults with type 2 diabetes.

## Appendix Table. Characteristics of Medicaid Beneficiaries With Diabetes in Alabama Overall, With ≥1 HbA_1c_ Claim, and With ≥1 HbA_1c_ Value, 2011–2019^a^

**Table:**

Characteristic	Overall (N = 262,310)	≥1 HbA_1c_ claim (n = 149,647)	≥1 HbA_1c_ value (n = 43,997)
**Age, mean (SD), y**	50.5 (10.3)	50.5 (10.3)	51.0 (9.9)
**Sex, no. (%)**			
Male	79,976 (30.5)	43,944 (29.4)	13,485 (30.6)
Female	182,334 (69.5)	105,703 (70.6)	30,512 (69.4)
**Race and ethnicity, no. (%)**			
Black	130,031 (49.6)	73,031 (48.8)	21,141 (48.1)
Hispanic	1,151 (0.4)	707 (0.5)	184 (0.4)
White	113,162 (43.1)	64,173 (42.9)	18,892 (42.9)
Other^b^	17,966 (6.8)	11,736 (7.8)	3,780 (8.6)
**Geography, no. (%)**			
Urban	166,832 (63.6)	94,855 (63.4)	29,816 (67.8)
Small town	39,090 (14.9)	23,265 (15.5)	6,318 (14.4)
Rural	56,388 (21.5)	31,527 (21.1)	7,863 (17.9)
**Reason for eligibility, no. (%)**			
Disability	133,398 (50.9)	100,728 (67.3)	34,197 (77.7)
Poverty	128,912 (49.1)	48,919 (32.7)	9,800 (22.3)
**Insulin use in prior year, no. (%)**	61,708 (23.5)	48,035 (32.1)	14,654 (33.3)
**Ambulatory care use**			
No. of primary care visits, mean (SD)	4.47 (3.17)	4.77 (3.04)	4.96 (2.95)
≥1 Primary care visit, no. (%)	232,234 (88.5)	137,649 (92.0)	41,363 (94.0)
No. of endocrinology visits, mean (SD)	0.11 (0.56)	0.14 (0.64)	0.10 (0.56)
>1 Endocrinology visit, no. (%)	13,056 (5.0)	9,301 (6.2)	2,025 (4.6)
**Acute care use, no. (%)**			
≥1 Emergency department visit	152,819 (58.3)	89,833 (60.0)	25,991 (59.1)
≥1 Hospitalization	84,559 (32.2)	48,145 (32.2)	13,141 (29.9)
**Medical comorbidities, no. (%)**			
Myocardial infarction	7,823 (3.0)	4,504 (3.0)	1,158 (2.6)
Congestive heart failure	36,749 (14.0)	21,480 (14.4)	5,929 (13.5)
Peripheral vascular disease	23,953 (9.1)	13,377 (8.9)	4,022 (9.1)
Cerebrovascular disease	29,609 (11.3)	16,591 (11.1)	4,467 (10.2)
Dementia	4,616 (1.8)	2,069 (1.4)	449 (1.0)
Chronic pulmonary disease	84,459 (32.2)	48,480 (32.4)	14,343 (32.6)
Rheumatic disease	8,822 (3.4)	4,858 (3.2)	1,261 (2.9)
Peptic ulcers	4,858 (1.9)	2,771 (1.9)	781 (1.8)
Diabetes with complication	71,154 (27.1)	44,162 (29.5)	12,316 (28.0)
Paraplegia and hemiplegia	6,822 (2.6)	3,247 (2.2)	788 (1.8)
Chronic renal failure	29,352 (11.2)	17,164 (11.5)	4,563 (10.4)
Mild liver disease	13,107 (5.0)	8,216 (5.5)	2,454 (5.6)
Moderate-severe liver disease	1,738 (0.7)	1,049 (0.7)	299 (0.7)
Any malignancy	11,589 (4.4)	6,590 (4.4)	1,892 (4.3)
Metastatic solid tumor	1,557 (0.6)	785 (0.5)	203 (0.5)
AIDS	2,789 (1.1)	1,561 (1.0)	420 (1.0)

Abbreviation: HbA_1c_, glycated hemoglobin A_1c_.

^a^ Data source: Enrollment and claims data for people with diabetes were provided for research purposes by the Alabama Medicaid Agency. HbA_1c_ values for Alabama Medicaid beneficiaries were provided for research purposes by LabCorp and Quest Diagnostics.

^b ^Includes Asian or Pacific Islander, American Indian or Alaskan Native, other race or ethnicity, or unknown/not provided.
